# Spatial Working Memory Deficits Represent a Core Challenge for Rehabilitating Neglect

**DOI:** 10.3389/fnhum.2013.00334

**Published:** 2013-06-27

**Authors:** Christopher L. Striemer, Susanne Ferber, James Danckert

**Affiliations:** ^1^Department of Psychology, Grant MacEwan University, Edmonton, AB, Canada; ^2^Department of Psychology, University of Toronto, Toronto, ON, Canada; ^3^Department of Psychology, University of Waterloo, Waterloo, ON, Canada

**Keywords:** neglect, spatial working memory, prism adaption, rehabilitation, parietal lobe

## Abstract

Left neglect following right hemisphere injury is a debilitating disorder that has proven extremely difficult to rehabilitate. Traditional models of neglect have focused on impaired spatial attention as the core deficit and as such, most rehabilitation methods have tried to improve attentional processes. However, many of these techniques (e.g., visual scanning training, caloric stimulation, neck muscle vibration) produce only short-lived effects, or are too uncomfortable to use as a routine treatment. More recently, many investigators have begun examining the beneficial effects of prism adaptation for the treatment of neglect. Although prism adaptation has been shown to have some beneficial effects on both overt and covert spatial attention, it does not reliably alter many of the perceptual biases evident in neglect. One of the challenges of neglect rehabilitation may lie in the heterogeneous nature of the deficits. Most notably, a number of researchers have shown that neglect patients present with severe deficits in spatial working memory (SWM) in addition to their attentional impairments. Given that SWM can be seen as a foundational cognitive mechanism, critical for a wide range of other functions, any deficit in SWM memory will undoubtedly have severe consequences. In the current review we examine the evidence for SWM deficits in neglect and propose that it constitutes a core component of the syndrome. We present preliminary data which suggest that at least one current rehabilitation method (prism adaptation) has no effect on SWM deficits in neglect. Finally, we end by reviewing recent work that examines the effectiveness of SWM training and how SWM training may prove to be a useful avenue for future rehabilitative efforts in patients with neglect.

One of the most debilitating disorders arising from right hemisphere brain damage is known as neglect. Neglect typically results from damage to the right temporal-parietal or superior temporal cortex (Vallar and Perani, 1986; Karnath et al., 2001, 2004; Mort et al., 2003; Buxbaum et al., 2004; Verdon et al., 2010; Karnath and Rorden, 2012), or from damage to subcortical structures such as the basal ganglia or thalamus (Karnath et al., 2002). Clinically, neglect is characterized by an inability to attend to or interact with people or objects on the contralesional (i.e., left) side (for reviews, see Heilman et al., 2002; Mesulam, 2002; Husain and Rorden, 2003; Danckert and Ferber, 2006). In severe cases, patients may act as if the left half of their world has simply ceased to exist (Mesulam, 1981). This unique, lateralized deficit of awareness for objects and events in the environment can greatly reduce the patient’s quality of life. Given that neglect is quite prevalent, occurring in 40–70% of all cases of right hemisphere stroke (Cherney and Halper, 2001; Buxbaum et al., 2004; Karnath et al., 2004; Ringman et al., 2004), and is a significant predictor of poorer overall functional recovery (Cherney et al., 2001), finding effective methods to rehabilitate the disorder is of great clinical importance.

Traditional models of neglect have focused on impaired spatial attention as the core deficit (e.g., Posner et al., 1984; Kinsbourne, 1993; Behrmann et al., 1997; Driver and Mattingley, 1998; Bartolomeo and Chokron, 2002). Specifically, neglect patients have been shown to have a rightward attentional bias (i.e., they preferentially attend to information on the right side). This is consistent with “gradient” models of neglect (Kinsbourne, 1987, 1993) which suggest that neglect severity increases for more leftward locations in space (i.e., even leftmost locations in right space are neglected more than locations further rightward). Neglect patients are also thought to have a “disengage deficit” such that they have great difficulty reorienting attention from right to left, neglected space (Posner et al., 1984; Bartolomeo and Chokron, 2002).

More recent studies have shown that neglect is a heterogeneous disorder comprised of a constellation of deficits including impaired temporal allocation of attention (Husain et al., 1997), poor time perception (Danckert et al., 2007; Merrifield et al., 2010; Oliveri et al., 2013), and spatial working memory (SWM) impairments evident throughout visual space (Husain et al., 2001; Ferber and Danckert, 2006). We will argue here that the deficits in SWM represent a core component of the disorder and as such, should be a target for rehabilitative strategies.

## Rehabilitating Neglect

Given that neglect is such a debilitating disorder, a great deal of research has focused on developing effective rehabilitation methods. A full analysis of each of these rehabilitation methods is beyond the scope of the current review (for a systematic review, see Luaute et al., 2006). Although many different techniques, including visual scanning training (Weinberg et al., 1977), caloric vestibular stimulation (Rubens, 1985), optokinetic stimulation (Pizzamiglio et al., 1990), neck muscle vibration (Karnath, 1995), and limb activation (Robertson and North, 1993) have been shown to have some benefits for neglect patients, most are impractical for a variety of reasons. For example, although visual scanning training has been shown to be effective in some studies (e.g., Weinberg et al., 1977, 1979), it typically involves a lengthy training program (from weeks to months) and requires the patient to make a conscious effort to attend to left space which is difficult given that many patients lack insight into their rightward bias. Techniques such as caloric vestibular stimulation, optokinetic stimulation, and neck muscle vibration, which induce a temporary nystagmus, can be uncomfortable for the patient, are challenging to implement on a regular basis, and typically only lead to a brief amelioration of symptoms (i.e., lasting only around 30 min; Rubens, 1985; Pizzamiglio et al., 1990; Vallar et al., 1990; Karnath, 1995). Finally, limb activation, in which the patient is encouraged to utilize their left, contralesional limb (Robertson and North, 1993; Robertson et al., 1995; Eskes et al., 2003), is impossible for the most severely hemiparetic, and impractical for other patients who now rely more heavily on their intact ipsilesional limb for whatever degree of independence they can achieve.

## Prism Adaptation and Neglect

**Figure 1:**
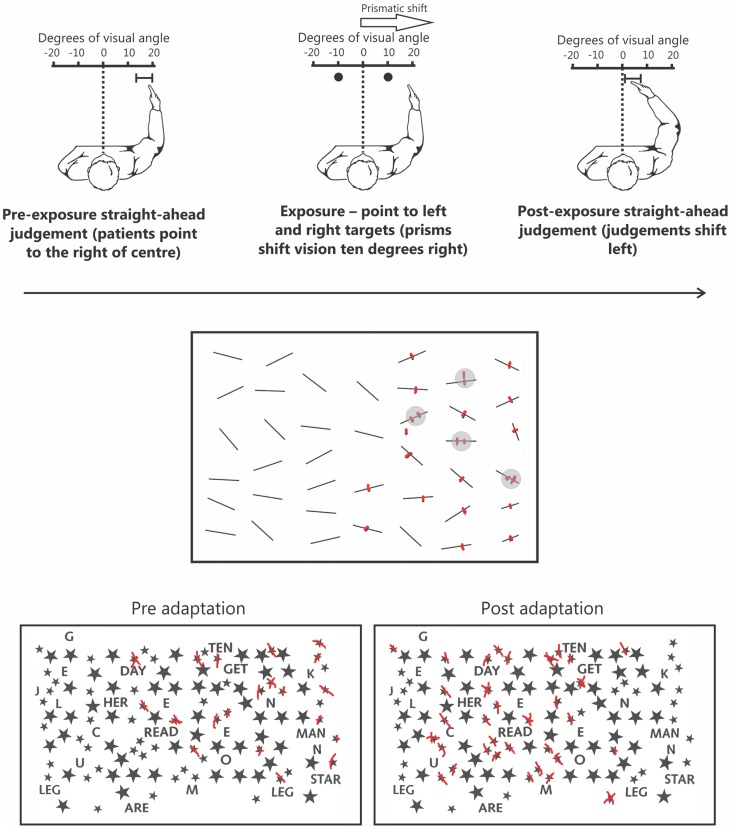
**The upper panel depicts the prism adaptation procedure used in neglect**. Left: prior to adaptation the patient is blindfolded and asked to point straight ahead of their body midline. Owing to an altered egocentric reference frame, patients typically point far to the right. Middle: during the adaptation procedure patients wear prisms that shift their vision 10° to the right. When asked to point to targets to the left and right they initially miss to the right because of the visual shift induced by the prisms. Right: following ∼5 min of prism adaptation, when the patient is again asked to close their eyes and point straight ahead, they now point much closer to true center. The middle panel depicts typical performance on a cancelation test. Specifically, in addition to missing numerous targets on the left side of the page the patient has also missed a target on the right side of the page. Note that the patient is also demonstrating “revisiting” behavior (highlighted by gray circles) by re-canceling previously canceled items as if they were new, indicative of impaired spatial working memory. The lower panel depicts an example of how prism adaptation improves performance on clinical tests of neglect. Prior to prism adaptation the patient misses targets on the left side of the page. However, following adaptation the patient now cancels many more targets on the left side of the page.

One rehabilitation technique that does not suffer from many of these same limitations, and has been shown to be reasonably effective, is the prism adaptation procedure developed by Rossetti et al. (1998). In this procedure, patients wear prismatic lenses that shift vision temporarily further rightward. While wearing prisms, the patient points to targets located to the left and right of their body midline. Initially, the patient misses to the right due to the visual shift induced by the prisms. Over successive trials the patient must make leftward corrections for their initial rightward pointing errors (for reviews of the prism adaptation method, see Redding et al., 2005; Redding and Wallace, 2006). After only a brief (∼5 min) exposure period, once prisms are removed, the patient now makes *leftward* pointing errors – the so-called after-effect. This after-effect is associated with a range of changes in behavior including exploratory behaviors that now shift leftward, into neglected space, and dramatic improvements on standard clinical tests of neglect (Figure 1; Rossetti et al., 1998).

Since this original study a plethora of studies have shown that prism adaptation can influence a broad range of neglect symptoms, with positive effects seen for spatial attention (Berberovic et al., 2004; Striemer and Danckert, 2007; Nijboer et al., 2008; Schindler et al., 2009), extinction (Maravita et al., 2003), exploratory eye movements (Dijkerman et al., 2003; Ferber et al., 2003; Angeli et al., 2004; Serino et al., 2006), posture and balance (Tilikete et al., 2001), and somatosensory function (McIntosh et al., 2002; Dijkerman et al., 2004). There is, however, also some controversy surrounding whether or not prisms lead to changes in the strong perceptual biases evident in neglect – biases that favor right space or the right half of objects (Dijkerman et al., 2003; Ferber et al., 2003; Sarri et al., 2006, 2010; Striemer and Danckert, 2010a,b). Specifically, some studies have demonstrated that while prisms can induce a leftward shift in exploratory motor behaviors and covert attention (Dijkerman et al., 2003; Ferber et al., 2003; Striemer and Danckert, 2010a), these changes do not necessarily translate into changes in perceptual biases, which are a hallmark symptom of neglect (for a review, see Striemer and Danckert, 2010b). For example, when viewing vertically aligned chimaeric faces (faces shown as smiling on one side and neutral on the other) neglect patients typically report the face smiling on the right as appearing happier (Mattingley et al., 1993). Prior to any intervention, it can be shown that patients only *look* at the right side of such faces. We showed that after prism adaptation exploratory eye movements now took in the left side of the chimaeric faces as well as the right side (Ferber et al., 2003). Importantly, the patient continued to report that the right-sided smiling face appeared to be happier even though prisms had shifted his exploratory eye movements leftwards (Ferber et al., 2003). This dissociation between altered actions and attention, coupled with unchanged perceptual biases, is not unique to faces (Dijkerman et al., 2003; Ferber and Danckert, 2006; Striemer and Danckert, 2010a).

In addition, whereas some studies have shown that repeated exposure to prisms creates long-term benefits for neglect (Frassinetti et al., 2002; Serino et al., 2006, 2009; Shiraishi et al., 2008), recent randomized control trials have failed to observe any clear evidence for long-term improvements (Nys et al., 2008; Turton et al., 2009).

In summary, while prism adaptation is clearly beneficial for reducing attentional biases in patients, it may not be effective at addressing all of the cognitive deficits present in neglect. For example, one domain that has not been explored to any great extent (at least to our knowledge) is the influence of prism adaptation on non-spatially lateralized deficits in neglect such as SWM (Husain et al., 2001; Ferber and Danckert, 2006), time estimation (Danckert et al., 2007; Merrifield et al., 2010), and sustained-temporal attention (Husain et al., 1997). There is some controversy as to whether these deficits should be considered core symptoms of neglect (Danckert and Ferber, 2006), or viewed merely as *exacerbating* factors (Husain and Rorden, 2003). Given that attentional deficits can be rehabilitated (to some degree), while other perceptual biases remain unchanged, it is at least plausible that non-spatially lateralized impairments play a more central role in the disorder (Danckert and Ferber, 2006). Nevertheless, it remains undisputed that current therapeutic approaches cannot be considered unequivocally successful.

One deficit that would be particularly devastating for neglect patients is the inability to keep track of spatial information over time (i.e., SWM). Specifically, while a strong tendency to focus attention on right space undoubtedly biases the patient’s initial exploratory behaviors, an inability to keep track of where one has *already attended* will mean that left space is rarely, if ever, explored.

## Spatial Working Memory

Working memory is conceptualized as a core cognitive skill that underlies human thought processes (for a review, see Baddeley, 2003). For example, studies have linked working memory capacity to general fluid intelligence (Engle et al., 1999), and attentional control (Kane et al., 2001; Cabeza et al., 2008). Working memory is typically defined as the ability to hold information online after it has been removed from view, and it is thought to have a limited capacity. The classic working memory model first proposed by Baddeley and colleagues (for recent reviews, see Baddeley, 2003, 2012) suggested that working memory functions could be fractionated into three primary components: a phonological loop important for auditory and verbal working memory, a visuospatial sketchpad important for storing visual and spatial information, and a central executive that flexibly allocates attentional resources to the separate storage systems (Baddeley, 2003, 2012). Over the years, there have been several revisions to the model. Most notably for the purposes of the present review, is the division of the visuospatial sketchpad into visual and SWM. Specifically, this distinction suggests that remembering the location of an object requires separate visual codes to remember the identity and location. Indeed, research has shown that it is possible to observe selective deficits in either visual or SWM following brain damage (e.g., Della Sala et al., 1999). However, it is important to note that although it is possible to dissociate performance on tests of visual and SWM, many patients present with deficits on both measures (Della Sala et al., 1999). For our purposes we focus specifically on the relationship between neglect and SWM; that is, the maintenance of spatial information over time.

Interestingly, previous brain imaging studies have noted that SWM and spatial attention are controlled by many of the same brain regions including both the frontal and posterior parietal cortices (for reviews, see Awh and Jonides, 2001; Corbetta et al., 2002; Wager and Smith, 2003; Cabeza et al., 2008; Ikkai and Curtis, 2011). Based on these findings, and the fact that SWM performance can be enhanced at attended locations (Awh et al., 1998), some have argued that spatial attention is required in order to “rehearse” and maintain information in SWM (Awh et al., 1998; Awh and Jonides, 2001; Theeuwes et al., 2009). However, more recent behavioral studies have shown that SWM performance is not always enhanced at attended locations (Belopolsky and Theeuwes, 2009). This suggests that while spatial attention and SWM clearly involve overlapping brain networks, it is possible to dissociate them from one-another. Given that spatial attention and SWM involve largely overlapping brain networks it is not surprising that lesions to right fronto-parietal regions, in addition to leading to neglect, are also likely to cause deficits in SWM (e.g. Vallar and Perani, 1986; Mattingley et al., 1998; Karnath et al., 2001; Mort et al., 2003; Sapir et al., 2007).

## Spatial Working Memory in Neglect

Some of the most common clinical tests used to assess neglect are cancelation tasks in which patients must “cross out” target items embedded within an array of distracters (Figure 1). Densely neglecting patients will cancel out many more targets on the right than on the left side of the page. Although this pattern of performance is considered a classic manifestation of disordered spatial attention in neglect, recent data has shown that this deficit reflects impaired SWM independent of attentional biases (Husain et al., 2001; Wojciulik et al., 2001, 2004). On cancelation tasks, in addition to missing targets on the left, patients often fail to cancel targets presented in right, putatively non-neglected, space (Figure 1; see Danckert and Ferber, 2006). This deficit is suggestive of an inefficient search strategy in which the patient has trouble keeping track of where they have previously searched. A more direct confirmation of a SWM deficit comes from “revisiting” behavior in which patients will re-cancel items they have already canceled in right space, thus treating “old” items as if they were “new” (Figure 1; Husain et al., 2001; Wojciulik et al., 2001, 2004).

Wojciulik et al. (2001) had a neglect patient perform a variety of cancelation tasks to explore the role of SWM. In the first, the patient used a salient marker to indicate cancelations, whereas the second version had them make “invisible” marks (i.e., canceling targets with a capped marker). The patient made many more re-cancelations (i.e., “revisiting” errors) for targets in right space in the invisible compared to the visible marks condition. Thus, without a highly salient marker indicating that the patient had already canceled the item, she continued to treat previously canceled items as “new.” These same findings were later confirmed in a larger group of patients (Wojciulik et al., 2004). Critically, studies have since demonstrated that revisiting errors were not simply a manifestation of perseveration, as a majority of cancelations were delayed revisits (i.e., cancelations of old targets occurring after other targets had been canceled; Parton et al., 2006).

Husain et al. (2001) had a neglect patient perform a variety of cancelation tasks while eye movements were monitored. Despite making an equal number of leftward and rightward saccades, the patient’s search was largely restricted to the right half of the display. In addition, the patient also demonstrated significant revisiting behavior by re-fixating many items in right space. Importantly, follow-up experiments with the same patient demonstrated that this revisiting behavior was directly influenced by working memory load. That is, when the total search display was reduced, or the number of possible target items was decreased, revisiting behavior was also significantly reduced. Furthermore, the patient’s revisiting behavior was positively correlated with the number of items missed on the *left* side of the display (see also Mannan et al., 2005).

A closely related concept that may explain SWM difficulties evident in neglect involves the updating of spatial locations across successive saccades (Duhamel et al., 1992a,b; Heide et al., 1995; Pisella and Mattingley, 2004; Vuilleumier et al., 2007; Vasquez and Danckert, 2008). The process of updating spatial locations across saccades is commonly referred to as saccadic remapping. Saccadic remapping is typically studied using the “double step” saccade task. In this task participants must saccade to successive targets presented in under 200 ms. Relying on retinal information alone would lead to an erroneous saccade to the second target. Instead, observers anticipate the sensory consequences of the first saccade, remap their internal representation of space accounting for those sensory consequences, and make an accurate saccade to the second target (Duhamel et al., 1992a). Patients with neglect commonly fail to accurately acquire the second target in a double step saccade task (Duhamel et al., 1992b; Heide et al., 1995; Pisella and Mattingley, 2004; Vuilleumier et al., 2007). Interestingly, saccadic remapping deficits in neglect have been shown to correlate with neglect severity as measured by standard clinical tasks (Vuilleumier et al., 2007).

Although saccadic remapping deficits might contribute to SWM deficits in search and cancelation tasks which by their nature require successive saccades to find targets (for reviews, see Pisella and Mattingley, 2004; Danckert and Ferber, 2006), other studies have demonstrated SWM impairments in neglect that are not easily explained by remapping deficits. For example, Malhotra et al. (2005) adapted the well-known Corsi Block Tapping test that is widely used to assess a participant’s “spatial span” (a measure of SWM; Kessels et al., 2000). In this task, the patient is required to recall a sequence of spatial locations tapped out on blocks by the experimenter. In their version, Malhotra et al. (2005) presented the spatial sequences on a computer screen by illuminating colored disks in a pre-determined order. Following the presentation of the spatial sequence, the patient was asked to tap out the sequence in the correct order. Importantly, targets were aligned vertically in central space to avoid any confound from spatial orienting deficits. Results indicated that neglect patients had a significantly decreased spatial span (*M* = 1.3 positions) compared to right brain damaged patients without neglect (*M* = 2.6), and both young (*M* = 3.5) and elderly (*M* = 2.6) controls. Notably, this impairment of SWM was observed even though stimuli were presented in central, presumably non-neglected space.

**Figure 2:**
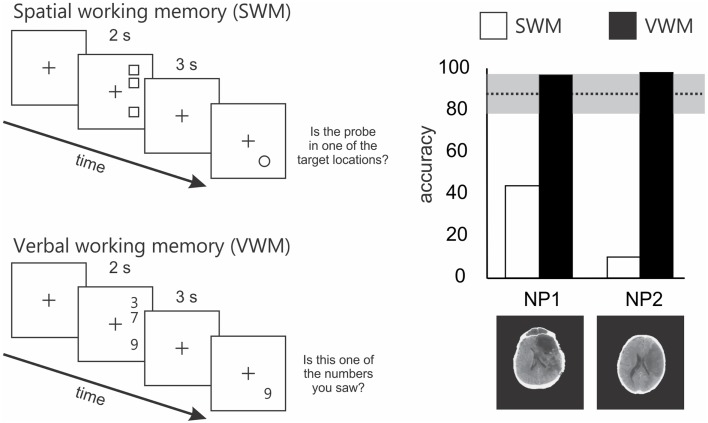
**Schematics of the spatial and verbal working memory tasks and results from 2 of the 4 patients tested by Ferber and Danckert (2006)**. The upper left panel depicts the layout of the spatial working memory task. Three squares were presented vertically aligned in right space for 2 s. Patients had to remember these locations over a 3-s delay. Following the delay a probe stimulus (a circle) appeared and the patient had to decide whether it was in a position previously occupied by one of the three squares. The lower panel depicts the layout for the verbal working memory task. Essentially the verbal working memory task used the same layout as the spatial working memory task. However, instead of remembering target locations patients had to remember three digits over a 3-s delay. Following the delay, the patient had to decide whether the probe digit was the same as one of the three previously presented digits. The right panel depicts the results of the spatial working memory task in a subset of two patients studied by Ferber and Danckert (2006). Specifically, both neglect patients performed extremely poorly on the spatial working memory task compared to right brain damaged controls (*n* = 4) without neglect (mean performance and standard deviation represented by the dotted line and gray bar). However, both neglect patients performed at ceiling on the verbal working memory task.

In a task similar to that used by Malhotra et al. (2005), we showed that the SWM impairment in neglect extended to right space (Ferber and Danckert, 2006). In our SWM task, target locations were vertically aligned in right space (Figure 2). On each trial patients were presented with three targets, followed by a brief delay (3 s). A circle probe then appeared and patients had to indicate whether the probe occupied a target location or not. Compared to right brain damaged patients without neglect and healthy controls, neglect patients were severely impaired on this task (Figure 2). Importantly, all groups performed at ceiling on a verbal working memory task that mirrored the spatial layout used in the SWM task (Figure 2). Thus, neglect patients do not suffer from a generic impairment of working memory, but instead demonstrate a domain specific problem related to SWM.

In summary, early studies indicated that neglect patients had difficulty keeping track of previously searched locations during cancelation tasks, suggestive of a deficit in SWM (Wojciulik et al., 2001, 2004). Subsequent studies extended these findings by demonstrating that SWM deficits were evident in neglect independent of spatial orienting deficits and when stimuli were presented in non-neglected space (Malhotra et al., 2005; Ferber and Danckert, 2006).

Given the overwhelming evidence implicating SWM deficits in neglect, any attempt to rehabilitate the disorder will be successful only inasmuch as it deals with this core deficit. Unfortunately, no studies to our knowledge have attempted to examine the effectiveness of current rehabilitation protocols for neglect on SWM deficits. In the next section, we will explore the effectiveness of prism adaptation, which could be considered the best treatment currently available for neglect, and its effects on SWM.

## Prism Adaptation and Spatial Working Memory in Neglect

As mentioned previously, a number of studies over the last decade suggest that prism adaptation can reduce both the rightward attentional bias and the “disengage deficit” which are prominent in neglect patients (Maravita et al., 2003; Berberovic et al., 2004; Striemer and Danckert, 2007; Nijboer et al., 2008; Schindler et al., 2009). In addition, prisms have also been shown to reduce exploratory motor biases such that patients begin to re-explore previously neglected (left) space (Dijkerman et al., 2003; Ferber et al., 2003; Serino et al., 2006); however, many of the perceptual biases remain unaltered following prism adaptation (Dijkerman et al., 2003; Ferber et al., 2003; Sarri et al., 2010; Striemer and Danckert, 2010a,b). In other words, although prism adaptation may mean that a neglect patient *can* attend more efficiently to the left in some circumstances, their residual perceptual biases mean that they are still *not likely* to attend to the left and/or that attended information may not reach the level of conscious awareness (for further discussion of this issue, see Danckert and Ferber, 2006). The fact that perceptual biases are largely unaffected following prism adaptation further reinforces the notion that neglect is much more than simply a disorder of attention and that many non-spatially lateralized deficits (including SWM deficits in central and right space) contribute significantly to the disorder. Therefore, it is our contention that a failure to address these non-spatially lateralized deficits will result in only a partial rehabilitation of neglect. Importantly, while the directional visuomotor remapping induced by prisms might be beneficial in helping patients attend to and explore previously neglected space, it is unclear what effect prisms might have on non-spatially lateralized deficits in neglect such as deficits in SWM (Striemer and Danckert, 2010b).

**Figure 3:**
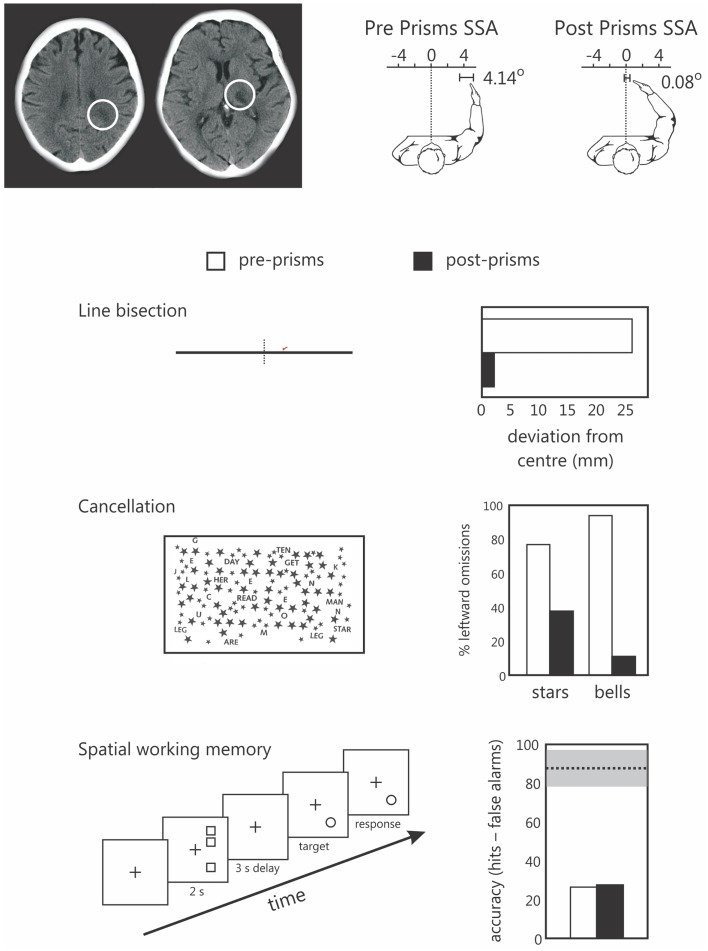
**Data from the single case study of patients NS, an 80-year-old right-handed female**. The upper panel depicts NS’s lesions to the parietal white matter (left) and thalamus (right) of the right hemisphere. To the right of these images are her subjective straight ahead (SSA) judgments made prior to prisms (left panel shows a 4.14° rightward bias) and after prism adaptation (SS = 0.08 degrees – not different from true center relative to her own body midline). The lower panels depict NS’s performance on line bisection, two cancelation tests, and the spatial working memory task prior to (pre-prisms; open bars), and following (post prisms; black bars) prism adaptation. Note that NS demonstrated a significant reduction in her rightward bias in line bisection, and a reduction in the number of items missed on the left in both cancelation tasks, but no change in her spatial working memory performance following prism adaptation. Note that for the spatial working memory data the dotted line and gray bar represent the mean performance (and standard deviation) of a group of right brain damaged controls without neglect tested in a previous study (Ferber and Danckert, 2006). We have since found a similar failure to improve SWM following prism adaptation in a group of six additional right brain damaged patients (Locklin and Danckert, in preparation).

We recently explored this in one neglect patient (patient NS) using our original SWM task in which targets are presented in right, putatively non-neglected space (Figure 3). Note that we have previously reported data from patient NS comparing the effects of prism adaptation on line bisection and landmark task performance (Striemer and Danckert, 2010a). Patient NS is an 80-year-old, right-handed female who presented with neglect (assessed via line bisection, cancelation, and figure copying) following a stroke affecting the right thalamus and surrounding white matter in right parietal cortex (Figure 3). Following prism adaptation patient NS demonstrated a significant leftward shift as measured by proprioceptive judgments of subjective straight ahead, that was evident by the end of the experiment (Figure 3). In addition, following prism adaptation, NS also demonstrated significant reductions in her rightward bias in line bisection, and an increase in the number of targets canceled on the left side of two cancelation tasks (Figure 3). Importantly, she showed no improvement whatsoever on our SWM task following prism adaptation (Figure 3). Note that in our previous study (i.e., Striemer and Danckert, 2010a) NS also failed to demonstrate any significant reduction in her rightward perceptual bias on the landmark task.

It is important to note that we have also found the same dissociation between beneficial effects of prisms on clinical tests of neglect but no changes in SWM performance in six additional right brain damaged patients, many of whom also had neglect (manuscript currently being prepared for publication).

Our contention that SWM performance is not altered by prism adaptation is further supported by a recent fMRI study by Saj et al. (2013) in which they examined performance in a bisection task, a spatial attention task, and a SWM task prior to and following prism adaptation in a group of seven neglect patients. Behavioral results indicated that prism adaptation improved performance on the bisection and spatial attention tasks, but did not improve SWM performance. Furthermore, their imaging results indicated that improvements in the bisection and spatial attention tasks were correlated with increased activity in the parietal, frontal, and occipital lobes bilaterally. However, no significant changes in activation were detected for the SWM task post prism adaptation.

Given that we are arguing that SWM represents a core deficit in neglect, one might question how prism adaptation can improve several aspects of neglect (i.e., exploratory motor biases, spatial attention) without influencing SWM? This is an important observation that we believe underscores two important points: (1) that it is *possible* to dissociate spatial attention from SWM performance (Belopolsky and Theeuwes, 2009); and (2) that neglect is a heterogeneous disorder comprised of a constellation of deficits and only by focusing on each of the deficits that comprise neglect will we be able to successfully ameliorate the disorder.

In summary, these results suggest that it is possible that prism adaptation may not be a effective treatment for SWM deficits in neglect, although further research in larger groups of patients is required for any definitive conclusions to be made. However, it is still important to highlight that even though research has clearly demonstrated that prisms can improve attention and exploratory motor behaviors, the research reviewed here suggests that it may not be effective for treating other aspects of neglect such as perceptual biases or SWM deficits. Therefore, developing new rehabilitation techniques that might reduce these additional components of neglect is necessary in order for a full recovery to occur. In the next section, we will discuss whether directed SWM training might be able to help further rehabilitate patients with neglect.

## Spatial Working Memory Training and Neglect

As mentioned previously, working memory can be considered a foundational cognitive skill that underlies human thought (Engle et al., 1999; Baddeley, 2003), and may serve as an interface between attention, perception, and decision making processes (Baddeley, 2003). It has also been shown that SWM in particular may rely, at least partially, on spatial attention in order keep spatial information active in memory when it is no longer visible (Awh et al., 1998; Awh and Jonides, 2001). Critically, both attention and SWM share common neural substrates in the frontal and posterior parietal lobes (for reviews, see Awh and Jonides, 2001; Wager and Smith, 2003; Husain and Nachev, 2007; Cabeza et al., 2008; Ikkai and Curtis, 2011). Therefore, damage to right hemisphere frontal and parietal cortex, regions shown to be involved in neglect (Vallar and Perani, 1986; Mattingley et al., 1998; Karnath et al., 2001; Mort et al., 2003; Sapir et al., 2007), will also result in severe deficits in SWM. Given that SWM is a foundational cognitive skill, any attempt to rehabilitate neglect must address this core cognitive deficit. Unfortunately, no current therapies for neglect directly address SWM as a target for rehabilitation. In addition, as just demonstrated, prism adaptation, which could be seen as one of the most promising rehabilitation techniques available for neglect may not have any influence on SWM capacity. Therefore, we would suggest that what is needed is a targeted therapy that focuses on retraining SWM in neglect.

One of the most important questions to address at the outset is whether it is actually possible to increase working memory capacity using training procedures. A series of recent studies suggest both that working memory capacity can be improved through training, and that such training may transfer to other cognitive capacities (Klingberg et al., 2002, 2005; Klingberg, 2010). This is not a trivial matter. One of the more persistent and recalcitrant challenges to rehabilitation and training in general is that improvement on the trained task often fails to lead to any improvement on untrained tasks (i.e., transfer).

Klingberg and colleagues (e.g., Klingberg et al., 2002, 2005) recently developed a computerized working memory training procedure using a variety of tasks (both verbal and SWM) that focus on increasing working memory capacity by adjusting the working memory load on a trial-by-trial basis based on the individual participant’s performance. Thus, the training procedure is tailored to the individual, and their current level of skill. Following the training regimen, participants demonstrate a significant improvement in working memory capacity as measured by the working memory training tasks (Klingberg et al., 2002, 2005; Westerberg et al., 2007). However, what is more impressive is the fact that the working memory training actually *transfers to untrained tasks* (Klingberg et al., 2002, 2005; Westerberg et al., 2007; Klingberg, 2010). That is, following training on a battery of verbal and SWM tasks, participants demonstrate improvements in other capacities including inhibition of unwanted responses (i.e., as measured by the Stroop; MacLeod, 1991), vigilance, and sustained attention (i.e., as measured by the continuous performance task and the paced auditory serial attention test; Beck et al., 1956; Tombaugh, 2006), SWM (as measured by other untrained tests), and reasoning (i.e., as measured by Raven’s progressive matrices). In other words, verbal and SWM training led to improvements in a broad range of cognitive skills that were not directly targeted by the training program itself (for a review, see Klingberg, 2010). Such improvements in working memory capacity and other cognitive abilities have been demonstrated in healthy individuals (Klingberg et al., 2002; Olesen et al., 2004), children with ADHD (Klingberg et al., 2002, 2005), and more recently, in stroke patients (Westerberg et al., 2007).

Interestingly, studies have shown that individual differences in visual working memory capacity in healthy individuals are positively correlated with activity in the intraparietal sulcus (e.g., Todd and Marois, 2004; Vogel and Machizawa, 2004). Olesen et al. (2004) examined which brain regions responded to SWM training by scanning healthy participants (using fMRI) before, during, and after 5 weeks of SWM training. The results indicated that SWM improvements following training were related to increased activity in the middle frontal gyrus and superior, inferior, and intraparietal regions bilaterally. This bilateral activation is important for the proposition being put forth here, namely that SWM training may help rehabilitate neglect. Specifically, any training related benefits may depend on the capacity for perilesional regions to be “retrained” and for homologous contralesional brain regions to compensate for lost function.

In summary, a number of studies have demonstrated that both verbal and SWM can be improved using training programs, and these improvements transfer to a variety of untrained tasks (Klingberg et al., 2002, 2005; Olesen et al., 2004; Westerberg et al., 2007; Klingberg, 2010). In addition, improvements in SWM capacity following training were shown to be positively correlated with activity in the middle frontal gyrus and posterior parietal cortex bilaterally (Olesen et al., 2004). Based on these data, SWM training in neglect may be expected to not only improve SWM capacity (a core deficit in neglect, Danckert and Ferber, 2006), but also to transfer to other untrained cognitive functions like attention (Westerberg et al., 2007), and executive control (Klingberg et al., 2002, 2005; Westerberg et al., 2007) which are also deficient in patients with neglect (e.g., Husain et al., 1997; Bartolomeo and Chokron, 2002; Danckert et al., 2011). Furthermore, SWM training might also be able to increase activity in undamaged regions of the frontal and posterior parietal cortex (Olesen et al., 2004) in the right hemisphere which are known to be chronically underactive in patients with neglect (Corbetta et al., 2005), as well as bootstrapping onto intact left hemisphere regions that may support retrained functions (Olesen et al., 2004).

Finally, it should be stated explicitly that we are not trying to suggest that SWM training alone will constitute a “cure” for neglect. It is quite conceivable that SWM training could be combined with other existing techniques that target more specific attentional and exploratory motor biases in neglect such as prism adaptation (and/or other techniques). In this sense we see SWM as being a complementary approach to many of the methods already in use to treat neglect.

## Conclusion

The evidence reviewed here suggests that SWM deficits are pervasive in neglect and thus constitute a core component of the syndrome. A severe limitation of the current strategies developed to rehabilitate neglect is that none of them specifically target SWM. What is needed then are rehabilitation strategies for neglect that are specifically aimed at increasing SWM capacity. The evidence reviewed here suggests that SWM training not only improves SWM performance, but also leads to improvements in untrained tasks (i.e., “transfer”; Klingberg, 2010). Furthermore, the improvements in SWM following training have been shown to rely on increased activity in frontal and parietal cortex bilaterally (Olesen et al., 2004). Therefore, we suggest that SWM training may constitute a promising avenue for future rehabilitative efforts in patients with neglect.

## Conflict of Interest Statement

The authors declare that the research was conducted in the absence of any commercial or financial relationships that could be construed as a potential conflict of interest.
